# The effect of *Ganoderma lucidum* spore oil in early skin wound healing: interactions of skin microbiota and inflammation

**DOI:** 10.18632/aging.103412

**Published:** 2020-07-21

**Authors:** Chunwei Jiao, Yizhen Xie, Hao Yun, Huijia Liang, Chunyan He, Aimin Jiang, Qingping Wu, Burton B. Yang

**Affiliations:** 1 College of Food Science, South China Agricultural University, Guangzhou 510642, P.R. China; 2 Guangdong Yuewei Edible Fungi Technology Co., Ltd., Guangzhou 510663, P.R. China; 3 State Key Laboratory of Applied Microbiology Southern China, Guangdong Provincial Key Laboratory of Microbial Safety and Health, Guangdong Institute of Microbiology, Guangdong Academy of Sciences, Guangzhou 510070, P.R. China; 4 Sunnybrook Research Institute, Toronto M4N 3M5, Canada; 5 Department of Laboratory Medicine and Pathobiology, University of Toronto, Toronto M5S 1A8, Canada

**Keywords:** wound healing, burn, microbiota, inflammation, LPS

## Abstract

The mushroom *Ganoderma lucidum (G. lucidum Leyss. ex Fr.) Karst* has been a traditional Chinese medicine for millennia. In this study, we isolated the *Ganoderma lucidum* spore oil (GLSO) and evaluated the effect of GLSO on skin burn wound healing and the underlying mechanisms. Mice were used to perform skin wound healing assay. Wound analysis was performed by photography, hematoxylin/eosin staining, Masson’s Trichrome staining and immunohistochemical analysis. Microbiota on the wounds were analyzed using the 16s rRNA sequence and quantitative statistics. The lipopolysaccharide (LPS) content was examined in skin wounds and serum using an enzyme-linked immunosorbent assay (ELISA). The expression of Toll-like receptor 4 (TLR4) and the relative levels of inflammatory cytokines were determined by qPCR and immunofluorescence assay. A pseudo-germfree mouse model treated with antibiotics was used to investigate whether GLSO accelerated skin burn wound healing through the skin microbiota. We found that GLSO significantly accelerated the process of skin wound healing and regulated the levels of gram-negative and gram-positive bacteria. Furthermore, GLSO reduced LPS and TLR4, and levels of some other related inflammatory cytokines. The assay with the pseudo-germfree mice model showed that GLSO had a significant acceleration on skin wound healing in comparison with antibiotic treatment. Thus, GLSO downregulated the inflammation by regulating skin microbiota to accelerate skin wound healing. These findings provide a scientific rationale for the potential therapeutic use of GLSO in skin burn injury.

## INTRODUCTION

Skin is the body’s largest and most exposed organ. It is the first line of the body’s immunological defense against various forms of attack from external environment [1]. Following skin injury, an organism needs to rapidly restore itself to avoid dehydration, blood loss and the entrance of harmful microorganisms [2]. Burns damage the skin extensively and have been studied for centuries, with thermal burns from dry sources (fire or flame) or wet sources (scalds) accounting for approximately 80% of all reported burn injuries [3]. Approximately 180,000 deaths annually are caused by burns; the vast majority occur in low- and middle-income countries [4]. Severe burn injury leads to a clear systemic inflammatory response that has been reported in human subjects [5, 6]. However, low public hygiene measures in low- and middle-income countries might lead to secondary bacterial infections. Therefore, antibiotics (ANT) for an anti-inflammatory effect are needed. Unfortunately, increased microbial drug resistance is being caused by widespread ANT use, leading to poor treatment efficacy in burn wound healing. Moreover, scar tissue formation after burn injury can also lead to long-term psychosocial consequences [7]. Therefore, effective and healthy treatments for burn wound healing are necessary for clinical therapy.

The mushroom *Ganoderma lucidum (G. lucidum Leyss. ex Fr.) Karst* (also known as Reishi or Lingzhi) has been one of the most intriguing traditional Chinese medicines for more than 2000 years and is documented by the Chinese Pharmacopoeia and Dietary Supplement Code of the United States Pharmacopoeia [8, 9]. The growth stages of *G. lucidum* include mycelium, fruit body and spore [10]. The fruit body of *G. lucidum* is the only part used for medical purposes in traditional therapy and has immunoenhancing and anti-inflammatory properties [11, 12]. Previous studies found that the extract of the *G. lucidum* fruit body had a clear effect on the acceleration of skin wound healing in mice and on human fibroblasts [13, 14]. In recent years, it has been reported that the spores of *G. lucidum* had significant effects on immunoregulation and anti-inflammatory activities [15, 16]. With the advance of sporoderm-breaking technology, more research has considered the biological effect of *G. lucidum* sporoderm-broken spores, in particular the *G. lucidum* spore oil (GLSO) that is extracted from *G. lucidum* sporoderm-broken spores [17]. However, little is known about the effect of GLSO on skin wound healing and the underlying mechanism.

Previous studies reported that *G. lucidum* was able to regulate the gut microbiota, which suggests the possibility to regulate other organismic microbiota [18]. A growing body of literature revealed that immunoregulation and inflammation are associated with organismic microbiota [19, 20]. Skin is colonized by various microorganisms, including bacteria, fungi and viruses as well as mites, and the skin microbial community has the potential to contribute to altered skin immune function [21]. Moreover, skin microbiota may affect wound healing through the pattern-recognition receptors (PRRs) [22]. Toll-like receptor 4 (TLR4), one of the PRRs, plays an important role in the innate immune system [23]. Understanding the role of TLR4 in the effect of skin microbiota on skin wound healing is important; however, few studies have focused on this area [24]. In the present study, we examined the effect of GLSO on the wound of a mouse burn model and its influence on skin microbiota abundance and the level of immunocytokines and related inflammatory cytokines, which revealed the underlying mechanism of GLSO on skin wound healing.

## RESULTS

### GLSO accelerated burn wound healing in ICR mice

**Figure 1:**
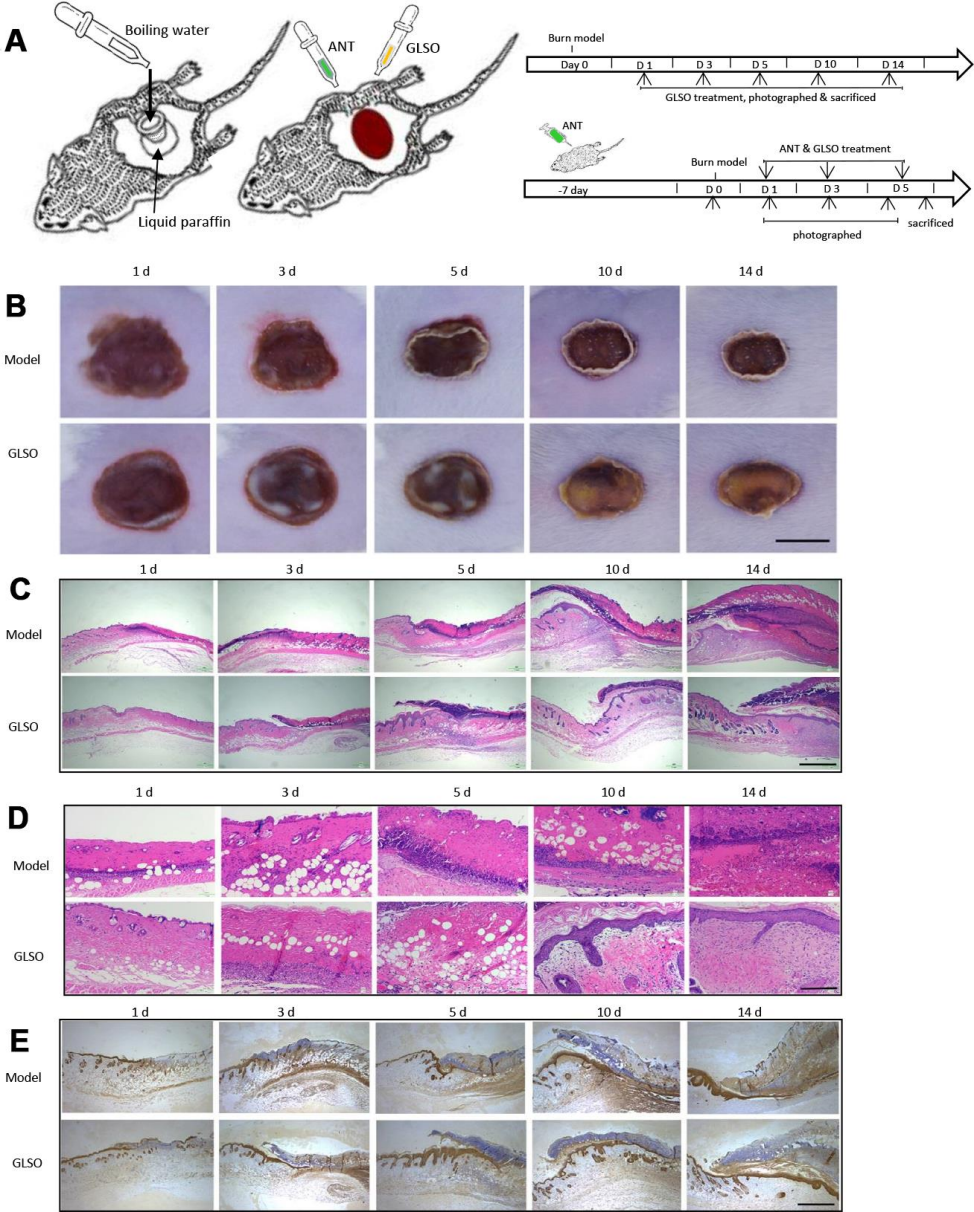
**GLSO accelerates scald wound healing.** (**A**) The experimental protocol of burn model and ANT treatment. (**B**) Gross examination of the wound area: a comparison of the model group with the GLSO group. Scale bar (**B**)=6 mm. (**C**, **D**) Images of H&E-stained tissue sections under the microscope 40X (**C**) and 200X (**D**) from the model group and GLSO group at different healing time points. Scale bar (**C**)=1 mm, scale bar(**D**)=200 μm. Immunohistochemical analysis of cytokeratin 14 under the microscope 40X (**E**) at the murine wound edge from the model group and GLSO group (scale bar=1 mm).

The burn model was used to investigate the role of GLSO in skin wound healing as shown (Figure 1A). From the images of the skin burn in mice, there was a greater healed area (white or yellow region) in the GLSO group compared with the model group (Figure 1B). H&E staining showed that necrotic cells had been replaced by new cells more rapidly in the GLSO group than in the model group throughout the 14-day treatment (Figure 1C, 1D). The immunohistochemical analysis of cytokeratin 14 at the murine wound edge showed that GLSO accelerated epidermal reconstruction of the burn wound in comparison with the model group (Figure 1E).

**Figure 2:**
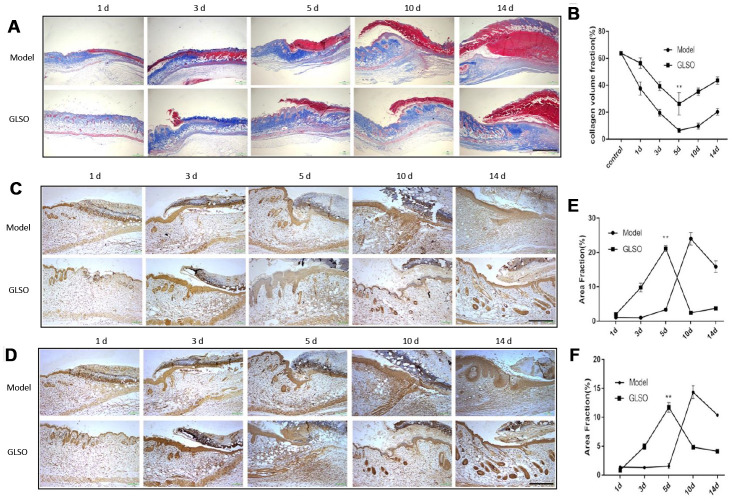
**GLSO has an obvious impact on the collagen deposition on day 5.** (**A**) Masson’s Trichrome was used to stain collagen deposition. Typical images of the stained tissue sections from the model group and GLSO group were from different healing time points under the microscope 40X (Scale bar = 1 mm). (**B**) Quantitation results of Masson’s Trichrome staining. (**C**, **D**) Immunohistochemical analysis of collagen I (**C**) and collagen III (**D**) at the murine wound edge from the model group and GLSO group under the microscope 100X (scale bar = 400 μm). (**E**, **F**) Quantitation results of collagen I (**E**) and collagen III (**F**) staining in comparison between the model group and GLSO group (n=3 each group).

Additionally, examination of collagen deposition at the murine wound by Masson’s Trichrome staining was performed (Figure 2A). The quantitative analysis revealed that GLSO increased collagen deposition before day 5 compared with the model group (Figure 2B). The result also showed that the collagen content of the skin wound in each group clearly increased after day 5. To define the impact of GLSO in transforming collagen deposition, immunohistochemical analysis of collagen I and collagen III was performed (Figure 2C, 2D). The quantitative results (Figure 2E and 2F) showed that GLSO increased the content of collagen I and collagen III more rapidly than the model group (both P < 0.01).

### GLSO decreased the content of inflammatory cytokines in early skin wound healing

**Figure 3:**
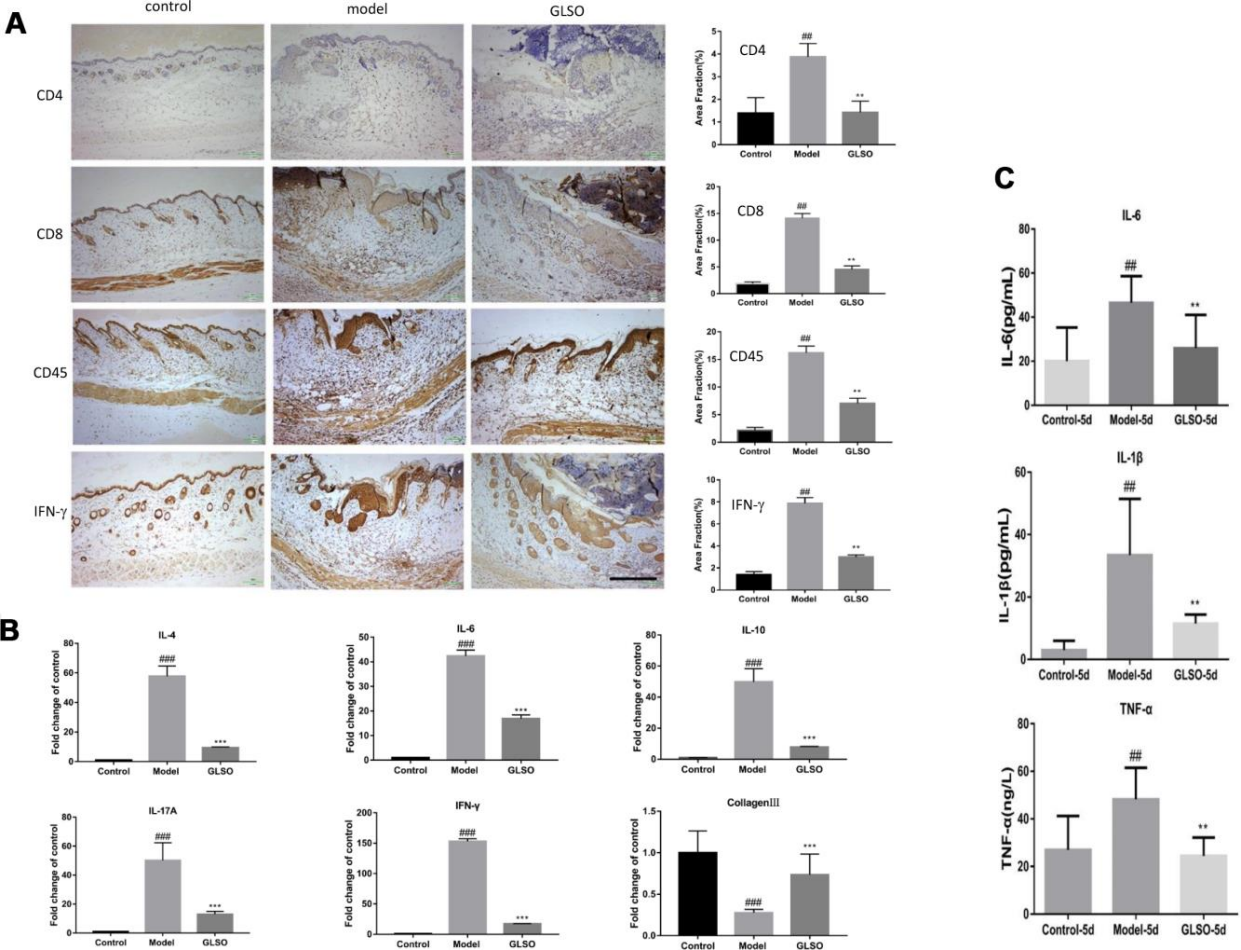
**GLSO decreases the level of inflammation.** (**A**) Immunohistochemical analysis of CD4, CD8 and CD45 at the murine wound edge on day 5 under the microscope 100X (scale bar=400 μm) was presented with quantitative analysis (n=3 each group). (**B**) The levels of inflammatory cytokines, IL-4, IL-10, IL-6, IL-17A and IFN-γ, were measured by real-time PCR. (**C**) The contents of IL-6, IL-1β and TNF-α were assayed by ELISA kit. The data are indicated as the mean±SD. ^##^*P* and ^**^P <0.01, ^###^*P* and ^***^P <0.001(^#^*P*: control *vs* model, ^*^P: GLSO *vs* model).

Immunostaining and quantitation analysis on day 5 showed that the GLSO group displayed significantly decrease in expression of CD4, CD8, CD45 and IFN-γ compared with the model group (Figure 3A, all p < 0.01). The results of the qPCR assay for IL-4, IL-6, IL-10, IL-17A and IFN-γ on day 5 demonstrated that the contents of these inflammatory cytokines was significantly reduced in the GLSO group in comparison with the model group (Figure 3B, all p < 0.001). The results of ELISA for IL-1β, IL-6 and TNF-α also showed that the GLSO group significantly reduced the levels of these inflammatory cytokines compared with the model group on day 5 (Figure 3C, all p < 0.01).

### GLSO had a significant impact on skin microbiota in skin wound healing

**Figure 4:**
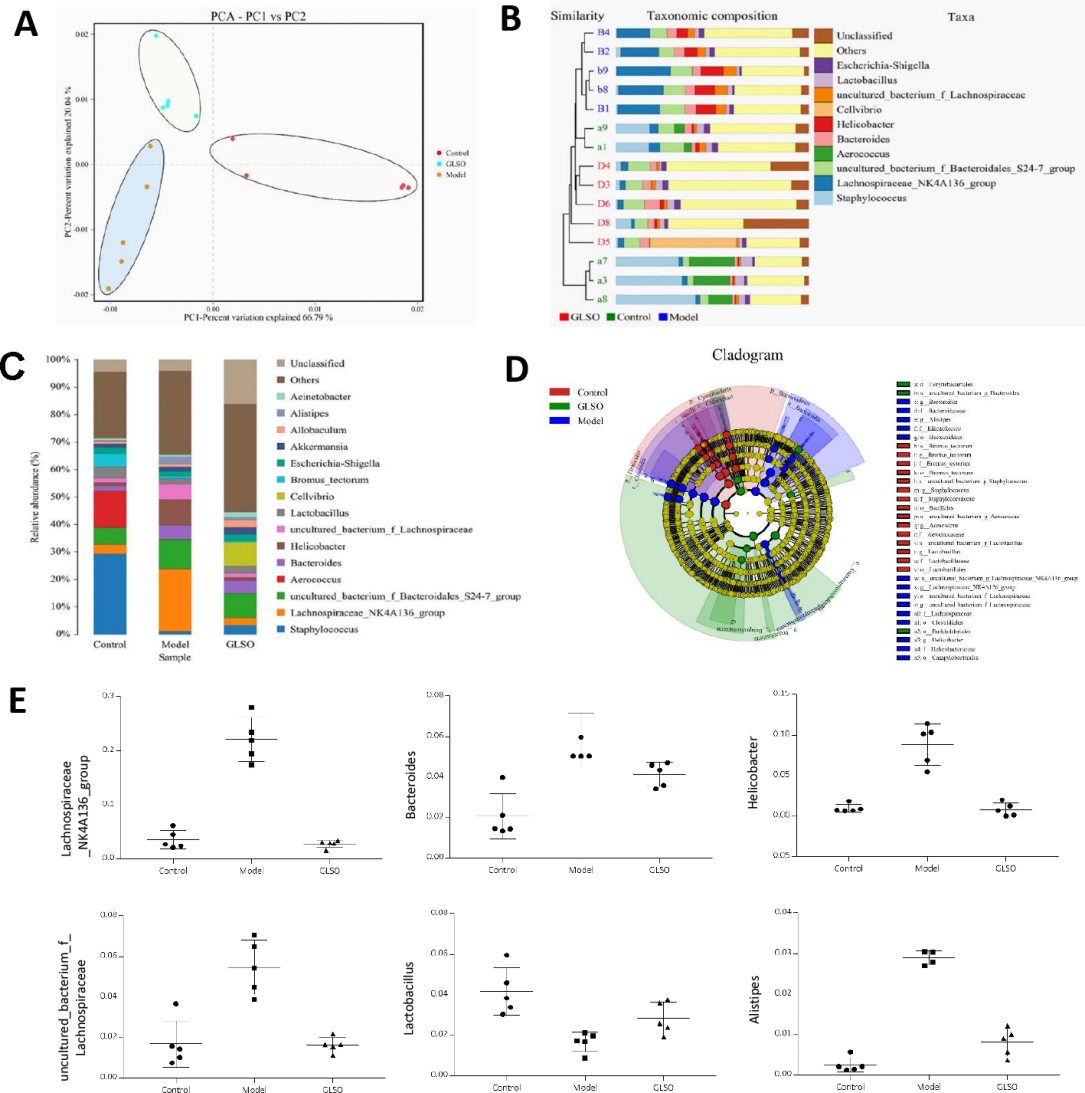
**Skin microbiota has a significant difference between the model group and GLSO group on day 5.** (**A**) Principal Component Analysis (PCA) which used variance decomposition to reflect the differences between multiple groups of data on a two-dimensional coordinate chart showed differences in clustering of microbial communities. (**B**) Unweighted Pair-group Method with Arithmetic Mean (UPGMA) analysis combined with histogram of species distribution in genus level was used to present the microbial similarities of each group and relative abundance. (**C**) Histogram of species distribution was used to compare the differences of relative abundance in genus levels between different groups. Line Discriminant Analysis Effect Size (LEfSe) analysis showed significant dominant bacteria on each group (**D**) and the abundance of six of these bacteria in genus levels (**E**). All analysis above were based on unweighted UniFrac.

The impact of skin wound microbiota on skin wound healing on day 5 was evaluated by 16S rRNA gene sequencing. Principal components analysis revealed that there was a clear difference in the microbial communities between the model and GLSO groups (Figure 4A). The similarity analysis was presented by using the taxonomic composition assay (Figure 4B). This analysis demonstrated that the taxonomic composition of the skin wound microbiota in the GLSO group was similar to the control group and GLSO displayed a clear difference compared with the model group. It was found that the relative abundances of *Alistipes*, *Lactobacillus*, *Helicobacter*, uncultured_ bacterium *f* *Lachnospiraceae,* *Lachnospiraceae*_NK4A136_group on GLSO treatment was clearly different compared with the model group by relative abundance analysis (Figure 4C). Lefse analysis showed the dominant bacteria in each group. The results showed that *Lachnospiraceae*_NK4A136_group, *Bacteroides*, *Helicobacter*, uncultured_bacterium_f_*Lachnospiraceae* and *Alistipes*, which are gram-negative bacteria, were significantly reduced with GLSO treatment compared to the model group (all P < 0.05) and the levels of these dominant bacteria on GLSO treatment was similar to the control group. Furthermore, *Lactobacillus* abundance was significantly reduced in the model group in comparison with the control group (P < 0.01) and increased in the GLSO-treated group (Figure 4D, 4E).

### GLSO downregulated the levels of LPS and TLR4 in early skin wound healing

**Figure 5:**
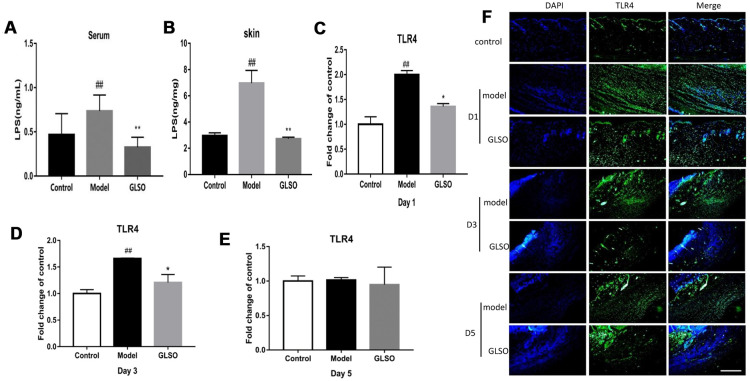
**GLSO downregulated the levels of LPS and TLR4 on early skin wound healing.** LPS levels in murine serum (**A**) and skin (**B**) was examined by the ELISA. The qPCR analysis revealed the levels of TLR4 at the murine wound edge on day 1 (**C**), day 3 (**D**) and day 5 (**E**). (**F**) Images of immunofluorescence from the model group and GLSO group at different healing time points were taken by immunofluorescent microscope and were compared with the control group under the microscope 200X. Scale bar = 200 μm. The data are indicated as the mean ± SD. ^##^*P* and ^**^P <0.01, ^###^*P* and ^***^P <0.001(^#^*P*: control *vs* model, ^*^P: GLSO *vs* model).

The strong reduction of gram-negative bacteria would lead to a decrease of endotoxin. ELISA examination of the LPS content in the skin and serum of the burn model was used to evaluate the endotoxin levels. The results revealed that GLSO treatment significantly decreased the LPS content in the skin and serum compared with the model group (both P < 0.01) (Figure 5A and 5B). The decreased LPS led to the reduction of TLR4. Immunofluorescence staining and qPCR analysis of TLR4 were used to indicate the differences between the model and GLSO groups on days 1, 3 and 5 (Figure 5C–5F). The results revealed that GLSO significantly decreased TLR4 on days 1 and 3 compared with the model group (both P < 0.05). However, there were no significant differences between the model and GLSO groups on day 5.

### GLSO accelerated skin wound healing more rapidly than ANT treatment

**Figure 6:**
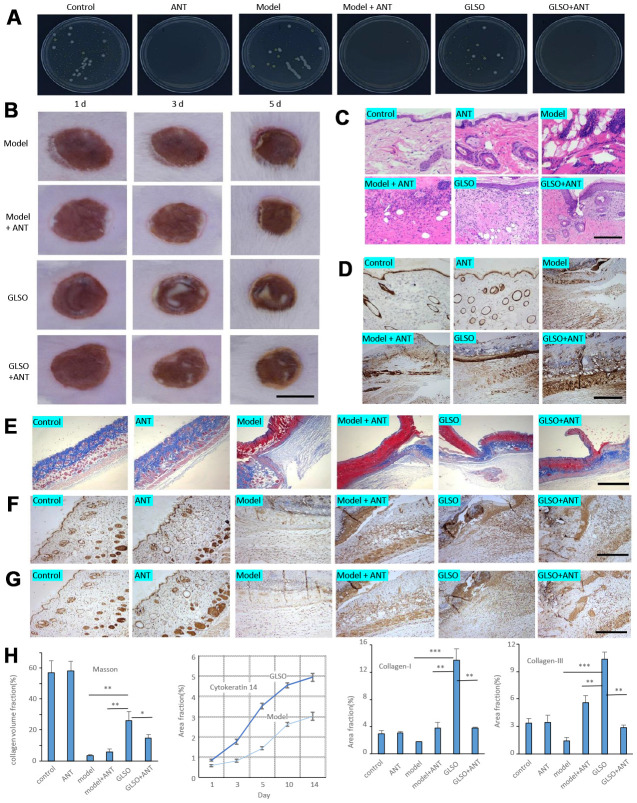
**GLSO accelerates the skin wound healing more rapidly than the group with treatment of antibiotics.** (**A**) The culture of skin microbiota on each group after 7 days with the treatment of antibiotics. (**B**) Gross examination of the wound area on each group. Scale bar =6 mm. (**C**) H&E staining of the wound tissue sections. (**D**) Immunohistochemical analysis of cytokeratin 14 under the microscope 100X, scale bar=400 μm. (**E**) Masson’s Trichrome staining, scale bar=1 mm. Immunohistochemical analysis of Collagen I (**F**) and Collagen III (**G**) at the murine skin wound on each group under the microscope 100X, scale bar=0.4 mm. (**H**) Quantitative analysis of Masson’s Trichrome staining, and immunostaining of cytokeratin 14 (quantitation results of Fig 1E), Collagen I, and Collagen III (n=3 each group). The data are indicated as the mean±SD. ^*^P<0.05,^**^P <0.01, ^***^P <0.001(^*^P: other groups *vs* GLSO).

**Figure 7:**
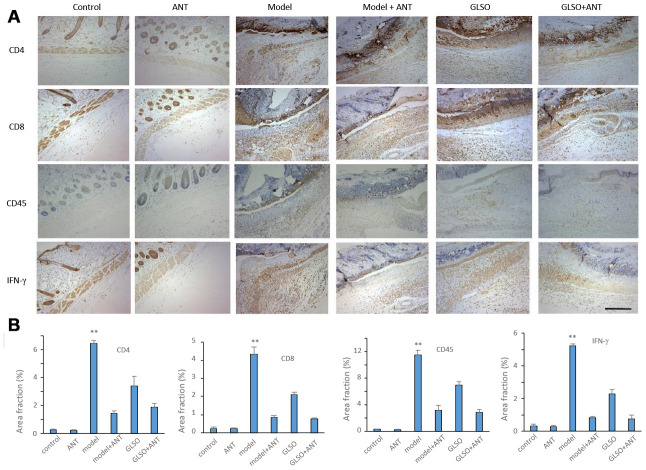
**GLSO accelerates the skin wound healing through the transform from inflammation to proliferation phase. **(**A**) Immunohistochemical analysis of relative inflammation cytokines under the microscope 100X. Scar bar=400μm. (**B**) Quantitative analysis of relative inflammation cytokines (n=3 each group). The data are indicated as the mean±SD. ^*^P<0.05, ^**^P <0.01, ^***^P <0.001(^*^P: other groups *vs* model).

To determine whether GLSO accelerated the process of skin wound healing through the skin microbiota, a pseudo-germfree mouse model was used. The culture of skin microbiota revealed that the ANT combination therapy had the ability to reduce the abundance of microbiota on murine skin (Figure 6A). From the images of burned skin in the mice, the healed area (white or yellow region) was greater in the GLSO group compared with the other groups (Figure 6B). The H&E staining (Figure 6C) and immunohistochemical analysis of cytokeratin 14 (Figure 6D) showed that the GLSO group accelerated the epithelialization of the skin wound more rapidly than the ANT and GLSO-ANT groups. The result of Masson’s Trichrome staining (Figure 6E) and immunohistochemical analysis of collagen I (Figure 6F) and collagen III (Figure 6G) as well as the quantitative analysis (Figure 6H) revealed that GLSO significantly increased the levels of collagen formation (all P < 0.01). However, in the assays of staining for CD4, CD8, CD45 and IFN-γ, we found that GLSO-ANT, ANT and GLSO groups all showed significantly decreased levels of these inflammatory cytokines relative to the of the model group (Figure 7A and 7B, all p < 0.01).

## DISCUSSION

Skin wound healing on burn injury is an extremely complex and dynamic process, traditionally including four overlapping main phases: hemostasis, inflammation, proliferation, remodeling. In the present study, GLSO accelerated the regeneration of the stratum corneum, which is considered to be an important marker for re-epithelization. Re-epithelization is an indicator for proliferation in skin wound healing and fibrous proteins such as collagens are formed during this phase [25–27]. Collagens are the main form of fibrous proteins and are organized into a partially cross-linked network by type I collagen (approximately 80%) and type III collagen (approximately 10%) [28]. GLSO promoted collagen formation and rapidly increased the content of type I and type III collagens, showing the important effect on fibrogenesis in wound healing. Moreover, the white or yellow region in the skin wound was found to recover the structure of normal skin, which further showed the GLSO acceleration of re-epithelization. Furthermore, the present study demonstrated that GLSO downregulated the levels of several inflammatory cytokines, showing a clear effect on the inflammation phase.

Inflammation has been reported to have an important influence in skin wound healing [29, 30]. The expression of inflammation after burn injury would lead to an increase in leukocytes. GLSO decreased the levels of CD4, CD8 and CD45 on the skin wound. CD45 is a marker for leukocytes, while CD4 and CD8 are markers specifically for lymphocytes, a leukocyte subtype and a reduction of leukocytes accelerated skin wound healing [31–34]. Increased lymphocytes and leukocytes on burn injury increased levels of pro-inflammatory cytokines, including IL-6, IL-1β and TNF-α [35]. As expected, GLSO decreased the IL-6, IL-1β and TNF-α levels of the wound. Interestingly, GLSO also reduced the levels of IL-4 and IL-10, which was reported to play an important role in delaying skin wound healing in their role as anti-inflammatory cytokines [36, 37]. The reduction of pro-inflammatory and inflammatory cytokines showed the effect of GLSO on the regulation of inflammation. Moreover, GLSO reduced IL-17A and IFN-γ expression after skin injury. IL-17A was reported to impair skin wound closure, while IFN-γ could decrease collagen formation and angiogenesis in wound healing [38–40]. Therefore, GLSO was able to accelerate skin wound healing by possibly regulating the relative levels of inflammatory cytokines.

Studies in recent decades have found that inflammation was associated with microbiota and skin microbiota was reported to influence skin wound healing [41]. Following skin injury, skin-resident microbiota and pathogenic species may colonize the wound and proliferate. This colonization and proliferation leads to bacterial infections that impair skin wound repair [42, 43]. The present study demonstrated that GLSO significantly decreased the levels of five genera of gram-negative bacteria and significantly aided recovery of the level of *Lactobacillus*, which showed that GLSO had the ability to regulate the genus level of skin microbiota. LPS was reported as a component of gram-negative bacterial cell wall, thus the addition of gram-negative bacteria increased the level of LPS, which was able to delay skin wound healing [44, 45]. The reduction of LPS in the skin wound and serum by GLSO confirmed that it could accelerate skin wound healing. Indeed, cutaneous cells respond to the foreign invasion, including bacterial infections, via the innate immune system. The skin innate immune system performs important functions, including the recognition and elimination of pathogenic organisms mainly via the skin PRRs, which include TLRs, and TLR4 plays a leading role among the TLRs [46]. High TLR4 expression leads to continuous inflammation, which can delay skin wound healing [47]. The present research demonstrated that GLSO decreased TLR4 expression during the early stage of skin wound healing. Indeed, LPS is a known inducer of TLR4, which indicated that GLSO downregulated the LPS levels by regulating skin microbiota and influenced TLR4 as well as inflammatory cytokines [48].

To confirm the effect of GLSO on skin wound healing by regulation of skin microbiota and inflammatory factors, a pseudo-germfree burned mouse model was used. The effect of ANT in the gut of the pseudo-germfree model was widely used to conduct in-depth research [49, 50]. Consequently, burn-injured mice with ANT treatment were used as a pseudo-germfree model. The present study showed that ANT significantly decreased the levels of all bacteria flora and the expression of inflammation. However, ANT had no significant effect on the repair of skin burn injury. In a review of studies of the anti-inflammatory effect of ANT, 45.71 % of the tested ANT accelerated skin wound healing, 34.29 % had no effect and 20% impaired skin wound healing [51]. The results above showed that ANT decreased the infiltration of inflammatory cells into the skin wound, but the effect on skin wound healing was uncertain. The present study also found that GLSO significantly accelerated skin wound healing in comparison with the GLSO-ANT group, which revealed the effect of skin microbiota on skin wound healing. Interestingly, the GLSO-ANT group also displayed significantly accelerated skin wound repair in the pseudo-germfree model compared with the model-ANT group. This indicated that the mechanism of GLSO in skin wound healing needs further investigation.

In summary, GLSO accelerated skin wound healing by down-regulating inflammation by regulating skin microbiota. Our previous research revealed that the main components of the glyceride in GLSO were 1,2-dioleoyl-3-palmitoyl-rac-glycerol, glyceryl trioleate, 1,2-dioleoyl-3-linoleoyl-rac-glycerol, 1-palmitoyl-2-oleoyl-3-linoleoyl-rac-glycerol and 1,2-dilinoleoyl-3-oleoyirac-glycerol [52]. However, the individual effect of these main components in skin wound healing is unknown and should be investigated. In the present study, we identified GLSO as an effective accelerant in skin wound healing in a burn wound model, which suggests its possible use in clinical application.

## MATERIALS AND METHODS

### Isolation of GLSO

Spores of *G. lucidum* were collected from the fruit body, which grew in the Dabie Mountain in An-Hui Province. The spores were treated with enzymes that are released from the spores themselves during different periods of growth using the GMP manufacturing facilities of Guangdong Yuewei Edible Fungi Technology Co. Ltd., Guangzhou, China. The oil-based fraction was extracted from the treated spores by supercritical fluid extraction (SFE). The soluble oil-based fraction was extracted in carbon dioxide fluid when the supercritical fluid of carbon dioxide was mixed with the treated *G. lucidum* spores. To isolate different fractions of the oil-based substance, the temperature and pressure were altered by the SFE machine. As part of the standard protocol for this study, the extraction was conducted at 40 ± 5 °C with a pressure of 25 ± 1 MPa. The carbon dioxide fluid velocity was 175 ± 15 L/h. The separating pressure was 6 ± 1 MPa, while the separating temperature was 45 ± 2 °C [53].

### Animals

All animal studies were conducted according to the Guidelines for the Use and Care of Laboratory Animals of the National Institutes of Health and the Animal Welfare Act Regulations. Each animal experiment was approved by the Ethics Committee of Guangdong Institute of Microbiology. All mice were purchased from Institute of Cancer Research (ICR) (Jinan Pengyue Experimental Animal Breeding Company, Shandong, China). Male mice at 7–8 weeks old, 32 ± 2.6g, were used for the studies. All mice were maintained in a constant environment (20–22 °C, 12-h light-dark cycle) with normal diet and were housed in individual cages to prevent further damage to the wounds. All experimental protocols were reviewed and approved by the Committee of the Guangdong Institute of Microbiology Animal Center (Permit Number: SYXK(YUE)-2016-0156).

### Burn wounding model

Mice were anesthetized with pentobarbital sodium (80 mg/kg) and shaved before burn wounding. A homemade device consisting of a hollow rubber tube was used in the present study. A circular wound was created by boiling water (1 ml) using the homemade device for 8 sec on the middle back of each mouse on day 0, with liquid paraffin applied to prevent overflow of boiling water around the homemade device (Figure 1A).

### Animal experiments

The mice were randomly assigned to three groups (n=15 for each): control, model and GLSO-treated. Mice, except for the control group, were anesthetized and shaved before burn wounding. Following wounding, 0.1 ml pure GLSO was applied on the wounds twice daily for up to 14 days in the GLSO-treated group. The mice were sacrificed on days 1, 3, 5, 10 and 14 (n=3 per group), photographing the wounds and collecting serum samples at these time points. Subsequently, the wounds were divided into three parts for further analyses (Figure 1A).

### Histology analysis

All tissues included normal skin tissues covering the wound edge were fixed with formalin and cut into 3-μm sections for hematoxylin/eosin (H&E) and Masson’s Trichrome staining following previous methods [25]. The percentage area of collagen deposition was calculated on the Image-pro Plus 6.0 (Media Cybernetics, Maryland, USA) by counting the number of clearly stained pixels and comparing with the total number of pixels. Images were captured using a microscope (Kangtao Technology, Wuhan, China).

### Enzyme linked immunosorbent assay (ELISA)

Blood and wound samples were collected and separated by centrifugation at 3500 rpm at 4 °C for 15 min. Interleukin-1β (IL-1β), IL-6 and tumor necrosis factor-alpha (TNF-α) levels were determined using Mouse IL-1β, IL-6 and TNF-α ELISA kits (Andy Gene, Beijing, China) according to the manufacturer’s instructions. The lipopolysaccharide (LPS) content of blood and wound samples were examined using Mouse LPS ELISA kits (Cusabio, Wuhan, China).

### Immunohistochemical analysis and immunofluorescence assay

Additional sections were deparaffinized through a graded series of dimethylbenzene and ethanol, and antigens were retrieved by incubation in citric acid buffer (pH 6.0, Abcam, Shanghai, China, ab93678) at 100 °C for 20 min. The sections were incubated with 3% H_2_O_2_ for 25 min to block endogenous peroxidase activity and then with 10 % goat serum albumin for 25 min to block nonspecific binding at room temperature.

Immunohistochemistry was performed as previously described [54, 55]. Briefly, the sections were incubated overnight at 4 °C with the following primary antibodies (all from Abcam) which were diluted in phosphate-buffered saline (PBS): CD45 (1:1000, ab10558), CD4 (1:500, ab183685), CD8 (1:200, ab203035), interferon gamma (IFN-γ) (1:500, ab9657), cytokeratin 14 (1:2000, ab181595), collagen I (1:50, ab34710) and collagen III (1:50, ab7778). Following washing with PBS, the sections were incubated with goat anti-rabbit immunoglobulin G (IgG) antibody (diluted in PBS, 1:1000, Abcam, ab205718) for 50 min at room temperature. The sections were washed again, incubated with 3,3-diaminobenzidine (Abcam, ab64238), washed and counterstained with hematoxylin. The sections were finally mounted with resin and imaged using a microscope (Kangtao Technology). In the positivity area, as the percentages of positive nuclei/total nuclei/field (at 100X magnification) in basal epidermal layer or dermal fibroblasts, was evaluated in at least three randomly selected fields/mouse. The mean score for every sample was calculated and analyzed. The inter-observer reproducibility was > 95%.

For immunofluorescence, the sections were incubated overnight at 4 °C with a primary antibody to TLR4 (diluted in PBS, 1:50, Abcam, ab13556). Following washing, the sections were incubated with goat anti-rabbit IgG immunofluorescence antibody (diluted in PBS, 1:200, Cell Signaling Technology, Boston, USA, 4412S) for 50 min at room temperature. The sections were washed again and incubated with 4',6-diamidino-2-phenylindole (DAPI, Servicebio, Wuhan, China, G1012) for 5 min at room temperature. Images were captured using an immunofluorescence inverted microscope (EVOS™ FL Auto 2 Imaging System, Shanghai, China).

### 16S rRNA gene sequencing analysis

Skin microbiota samples were collected in sterile tubes on day 5 and stored at −80 °C. Total DNA from skin microbiota samples was PCR amplified using primers targeting regions flanking the variable regions 3 through 4 of the bacterial 16S rRNA gene (V3–V4), gel purified and analyzed using multiplexing on HiSeq (Illumina, San Diego, USA). The amplification of a 416–425 bp sequence in the variable region V3–V4 of the 16S rRNA gene was performed using barcoded primers. The raw amplicon data were preformed using quantitative insights into microbial ecology.

### Real-time quantitative PCR (qPCR) analysis

Real-time PCR was performed as previously described [56–58]. In brief, total RNA was isolated from tissues using Trizol and chloroform. The quantity and quality of the RNA were analyzed using a NanoDrop One Microvolume UV-Vis Spectrophotometer (Thermo Fisher Scientific, Shanghai, China). Total RNA (2 μg) was transcribed with an iScript cDNA synthesis kit (TaKaRa, Dalian, China). qPCR was performed using SYBR PCR master mix (Applied Biosystems/Thermo Fisher Scientific) and a QuantStudioTM 6 Flex Real-time PCR system (Thermo Fisher Scientific). The PCR conditions were 40 cycles of 5 sec denaturation at 95 °C, 30 sec annealing at 55 °C and 5 sec extension at 65 °C. Glyceraldehyde-3-phosphate dehydrogenase was measured as a comparative reference. The primer sequences for qPCR are listed in PubMed primer blast online (Table 1). The mRNA relative quantitation was calculated using the ΔΔCt method.

**Table 1:** qRT-PCR primer sequences.

**Gene**	**Organism**	**Sequence 5'-3'**
*GAPDH*	Mouse	FW:AGGTCGGTGTGAACGGATTTG
		RV:TGTAGACCATGTAGTTGAGGTCA
*IFN-γ*	Mouse	FW:GAACTGGCAAAAGGATGGTGA
		RV:TGTGGGTTGTTGACCTCAAAC
*IL-4*	Mouse	FW:GGTCTCAACCCCCAGCTAGT
		RV:GCCGATGATCTCTCTCAAGTGAT
*IL-6*	Mouse	FW:TAGTCCTTCCTACCCCAATTTCC
		RV:TTGGTCCTTAGCCACTCCTTC
*IL-10*	Mouse	FW:GCTCTTACTGACTGGCATGAG
		RV:CGCAGCTCTAGGAGCATGTG
*IL-17A*	Mouse	FW:CTCCAGAAGGCCCTCAGACTAC
		RV:GGGTCTTCATTGCGGTGG
*Collagen III*	Mouse	FW:CTGTAACATGGAAACTGGGGAAA
		RV:CCATAGCTGAACTGAAAACCACC
*TLR4*	Mouse	FW:ATGGCATGGCTTACACCACC
		RV:GAGGCCAATTTTGTCTCCACA

### Pseudo-germfree mice model and treatment

A burn wounding model treated with ANT was used for the experiment. The mice were randomly assigned to six groups (n=6 for each): control, ANT, model, model-ANT, GLSO and GLSO-ANT. The groups with ANT received a combination therapy on the exposed back, which included ampicillin (10 mg/ml), neomycin (10 mg/ml), vancomycin (2 mg/ml) and metronidazole (10 mg/ml). Following 7 days of ANT combination treatment, burn wounding was performed on each mouse except for the control and ANT groups. 0.1 ml pure GLSO was applied on the wounds twice daily for 5 days in the GLSO and GLSO-ANT groups. The ANT combination therapy was also applied once daily on the wounds in the ANT, model-ANT and GLSO-ANT groups before the treatment with GLSO. The wounds were photographed on days 1, 3 and 5 and the mice were sacrificed on day 5 (Figure 1A). The wounds were divided into two parts for further analyses.

### Statistical analysis

All results were expressed as the mean ± standard deviation. Normal distribution and statistical comparisons between groups were determined by one-way analysis of variance and least-significant difference test using SPSS 19.0 (IBM, NY, USA). Graphs for statistical analysis were generated using GraphPad Prism 7, version 7.00 (GraphPad Software, San Diego, USA). For all statistical tests, the variance between each group was defined using the probability value P and values of P < 0.05 were considered to be statistically significant.
